# Pulmonary Metastasis from Pseudomyxoma Peritonei

**DOI:** 10.1155/2012/690256

**Published:** 2012-07-12

**Authors:** Toshiyuki Kitai

**Affiliations:** Department of Surgery, Kishiwada City Hospital, 1001 Gakuhara-cho, Kishiwada, Osaka 5968501, Japan

## Abstract

Pseudomyxoma peritonei (PMP) is a rare clinical condition, where copious mucinous ascites accumulate in the peritoneal cavity due to dissemination of mucin-producing tumor. Because of this disseminating, yet nonmetastasizing, behavior, PMP attracts much interest from surgical oncologists in that aggressive locoregional therapy can give the opportunity of long survival and even cure. Although extra-abdominal metastasis is exceptionally rare, the lung is the most likely site in such a case. In this paper, the clinical findings and treatment of eleven cases with pulmonary metastasis from PMP were reviewed, including ten cases in the literature and one case which we experienced. The clinical features of PMP cases with pulmonary metastasis were similar to cases without pulmonary metastasis. The histological type was low-grade mucinous neoplasm in most cases. Pulmonary lesions were resected in seven cases in which abdominal lesions were controlled by cytoreductive surgery and hyperthermic intraperitoneal chemotherapy or another therapeutic modality. Disease-free state was maintained in five cases at the end of the follow-up period. However, it should be noted that rapid progression after resection was seen in two cases, suggesting that biological features may have changed by surgical intervention.

## 1. Introduction

Pseudomyxoma peritonei (PMP) is a rare clinical condition, where copious mucinous ascites accumulate in the peritoneal cavity due to dissemination of mucin-producing tumor [1]. It is initiated by perforation of low-grade mucinous appendiceal neoplasm in most cases. Although the condition becomes fatal if untreated, progression is slow, and extra-abdominal metastasis is exceptionally rare. Because of these biological behaviors, PMP attracts much interest from surgical oncologists in that aggressive locoregional therapy can give the opportunity of long survival and even cure [2, 3]. Cytoreductive surgery (CRS) and hyperthermic intraperitoneal chemotherapy (HIPEC), which were initiated by Sugarbaker, have been accepted as an option for the standard treatment for PMP in many specialized centers.

Although extra-abdominal metastasis is very rare, the lung is one of the most probable sites in such a case. Once pulmonary metastasis occurs, it is important to consider that the biological features of PMP with metastasis may be different from those without metastasis. It is not clear whether surgical resection of metastatic lesions improves prognosis. In addition, if the biological features of PMP cases with pulmonary metastasis are more aggressive than those without pulmonary metastasis, CRS and HIPEC may not be indicated for these cases. In this paper, the clinical findings and treatment of eleven cases with pulmonary metastasis from PMP were reviewed, including ten cases reported in the previous literature and one case which we experienced.

## 2. Case

**Figure 1:**
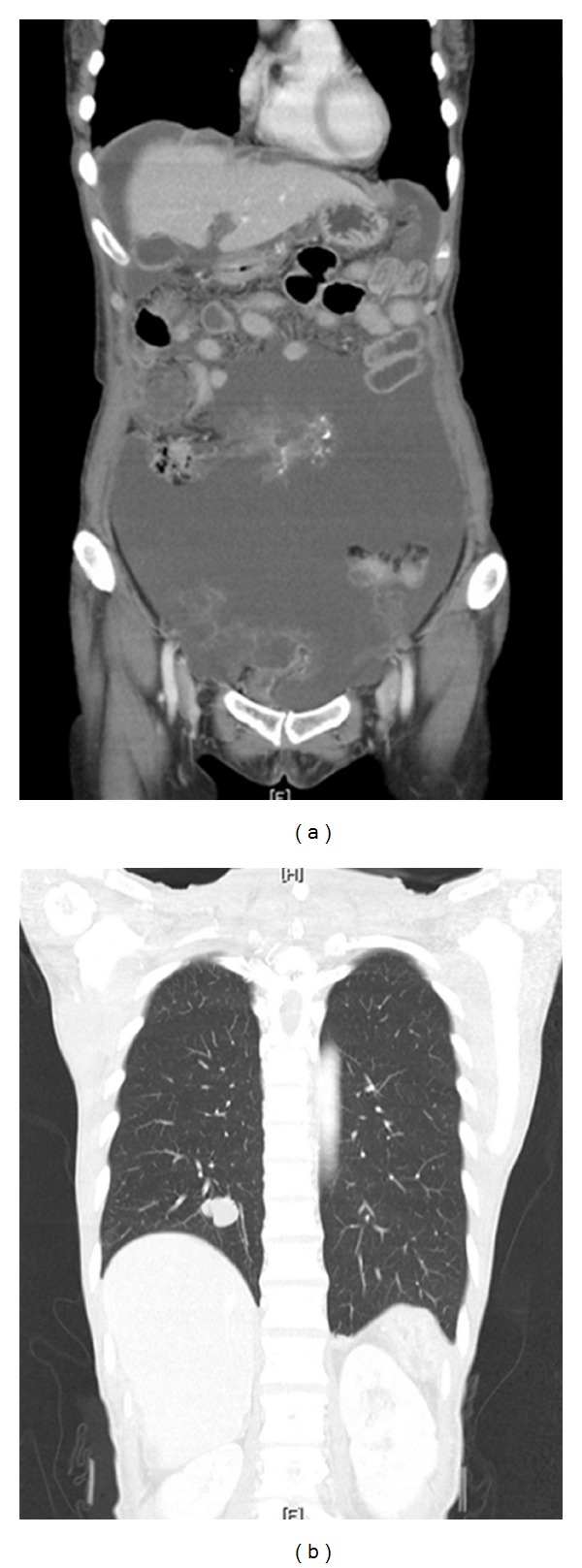
CT at the time of referral: massive tumor was diffusely spread in the peritoneal cavity. A solitary nodule in the right lower lung was also observed.

**Figure 2:**
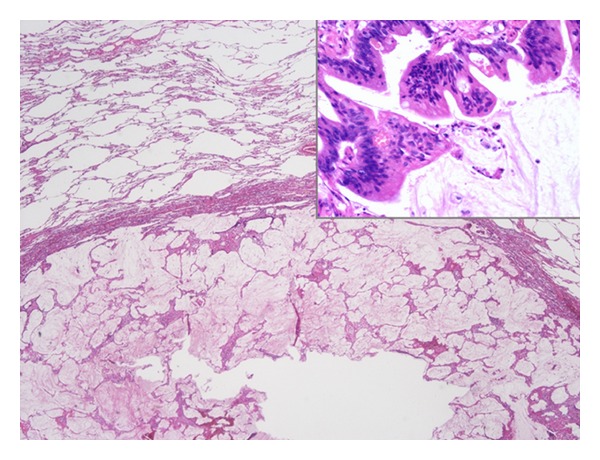
Histological findings of the lung nodule: atypical cells with histological characteristics similar to appendiceal tumor invaded pulmonary parenchyma.

**Figure 3:**
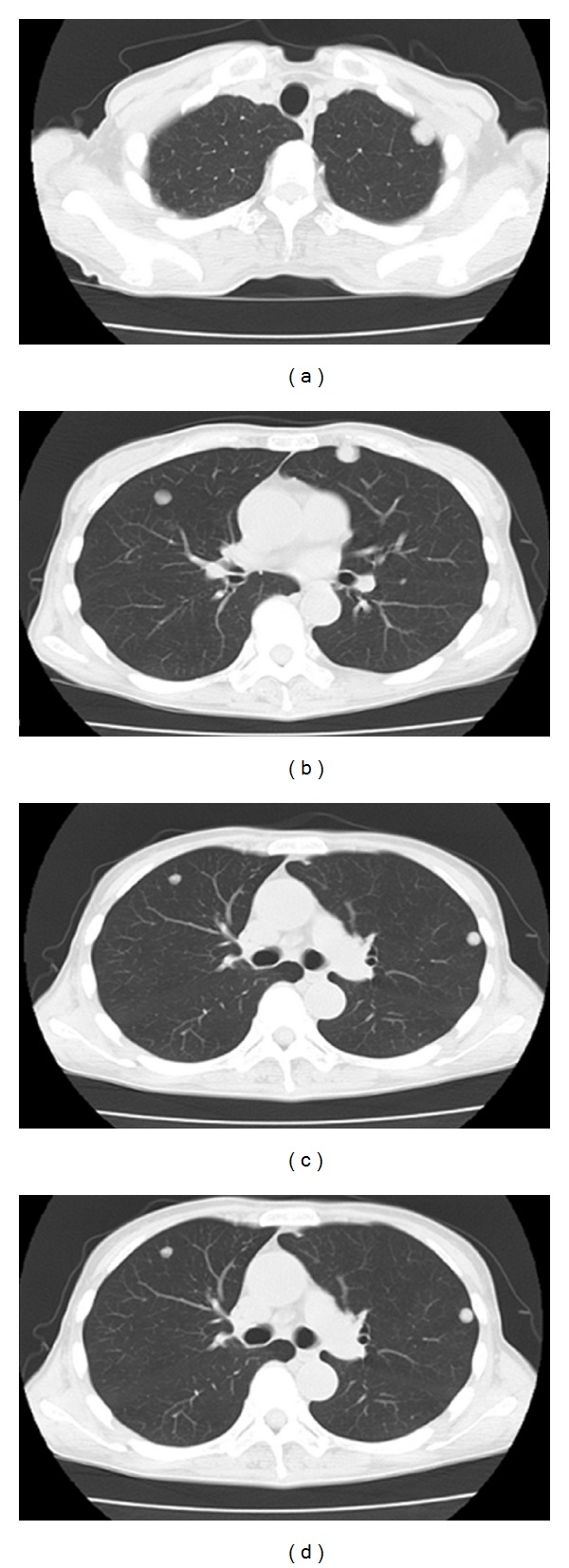
CT examination two months after CRS revealed rapid progression of multiple lung metastases.

A 60-year-old female was referred to us for the treatment of PMP. She had undergone palliative resection and HIPEC for PMP one year before. Histological diagnosis was low-grade appendiceal mucinous neoplasm with peritoneal dissemination, classified as disseminated adenomucinosis (DPAM) according to the criteria by Ronnett. At the time of referral, tumor was diffusely spread in the peritoneal cavity and single nodule was observed in the right lower lung (Figure 1). CRS and HIPEC were performed, and complete cytoreduction was achieved. The lung nodule was removed by wedge resection. Histological findings of the lung nodule were similar with those of appendiceal tumor, showing that low-grade mucinous neoplasm invaded pulmonary parenchyma (Figure 2). CT examination two months after CRS showed multiple lung nodules, and they progressed rapidly (Figure 3). She underwent laparotomy for intestinal obstruction caused by diffuse abdominal recurrence five months later. The histological type of recurrent lesions was the same as that of previously resected specimens. Serum levels of CEA and CA19-9 were 10.6 ng/mL and 62.3 U/mL before CRS, and returned to normal ranges after CRS, respectively. They still remained in the normal range at the time of CT examination, and increased again to 6.8 and 105.5 at the time of laparotomy. She died of the disease one year after CRS.

## 3. Review of the Literature

### 3.1. Clinical Findings

Eleven cases of pulmonary metastasis from PMP have been reported including the present case [4–10] (Table 1). Patients included seven males and four females, and the mean age was 51.8 years old (range: 39–65 years old). The origin of the disease was low-grade appendiceal neoplasm [11], and the histology of PMP corresponded to disseminated adenomucinosis [12] in most cases, although it is difficult to be sure from the description by some authors exactly how pulmonary metastasis would be classified. Two cases reported by Lee et al. [8] and Kahn et al. [10] were classified as well-differentiated mucinous adenocarcinoma and mucinous cystadenocarcinoma, respectively. However, from their description and published photographs, it would likely be considered as DPAM in both cases. Pulmonary metastasis was multiple in seven cases and bilateral in five cases. All cases were metachronous, and the median interval between the first clinical presentation of PMP and lung metastases was three years (range: 3 months–7 years). Pleural extension coexisted in two cases, but they were separated from pulmonary lesions.

### 3.2. Treatment

In the three cases which were reported earlier by Berge, Chevillotte et al., and Kreissig et al. [4–6], patients underwent palliative debulking surgery for PMP, and lung metastasis was histologically confirmed by autopsy or lung biopsy. CRS and HIPEC were performed in six out of the eight cases, which were reported more recently [7, 9]. Only CRS was done in one case [8], and appendectomy with radiotherapy was done in another case [10]. Pulmonary lesions were resected in the seven cases in which abdominal lesions were controlled by CRS and HIPEC or by appendectomy with radiotherapy. Wedge resection was done in four cases, and lobectomy with or without lymph node dissection was done in three cases to achieve disease-free state. Recurrence occurred in four cases, among which recurrent sites were the lung in three cases and the abdomen in one case. The mode of pulmonary resection did not affect the probability of recurrence. Intervals between pulmonary resection and recurrence were 2 months, 1 year, and 13 years in three cases. In one case, multiple pulmonary recurrences occurred shortly after resection, but the interval length was not exactly described. Salvage surgery for recurrent lesions was performed in two cases: one was CRS and HIPEC for abdominal recurrence, and the other was pulmonary wedge resection for pulmonary recurrence. Disease-free state was maintained in five out of the seven cases at the end of the follow-up period ranging from 2 to 14 years. However, two cases showed rapid progression after pulmonary resection and were judged as inoperable. There was no description in any report that systemic chemotherapy was performed for pulmonary metastasis from PMP.

## 4. Discussion

Extra-abdominal metastasis of PMP is exceptionally rare, but the lung and pleura are the most likely sites in such a case [9]. The majority of pleural metastases were caused by diaphragmatic injury at previous cytoreductive surgery or direct invasion through the diaphragm [13, 14]. Congenital pleuroperitoneal communication was also reported as a rarer cause [15]. They are thought to be extensions of dissemination rather than metastasis. By contrast, pulmonary metastasis is thought to occur through lymphatic fluid or venous blood. Although several cases of splenic metastasis have been reported as hematogenous, most lesions were thought to be an entrapment of mucinous tumor within splenic surface trabeculae, which expand into splenic parenchyma resembling metastatic disease [16]. Two cases showed coexistence of pleural extension in this review, but pulmonary lesions were separated from pleural lesions. A recent biological study reported that the decreased expression of E-cadherin and increased expression of N-cadherin and vimentin in tumor cells of PMP were more significant than in those of adenocarcinoma of the colon. The authors suggested that these specific phenotypes may characterize the disseminating, yet nonmetastatic, behavior of PMP [17]. It was also shown that Ki-67 expression significantly increased in adenocarcinoma but was similar in PMP as compared to that in normal colonic mucosa, suggesting a correlation with the slow growing behavior of PMP. Such specific biological features of PMP may have changed in pulmonary metastasis cases. Although no biological study was done in previous case reports, the histological types of PMP and pulmonary metastasis were classified as DPAM and low-grade mucinous neoplasm in all cases. PMP was well controlled by CRS and HIPEC in most cases. There seems to be no clinical finding suggesting that they had different biological features. However, as to the two cases which showed rapid progression after CRS and pulmonary resection, it was highly suspected that inflammatory reactions caused by surgical stress and other factors may have changed biological features which cannot be determined by the histological type.

Resection of metastatic lesions was indicated, when abdominal lesions were controlled by CRS and HIPEC. Wedge resection would be enough except such occasions where the possibility of primary lung cancer cannot be excluded. Prognosis was fairly good, although the follow-up period was rather short. It is noteworthy that long survival was achieved in one case in which multistep pulmonary resection was performed for multiple and bilateral lung metastases. However, it should be cautiously noted that rapid progression after pulmonary resection was seen in two cases. It was suspected that inflammatory reaction caused by CRS and abdominal infection may have changed biological features towards rapid deterioration [18, 19].

A more recent retrospective study showed that 42 cases of intrathoracic metastases were found out of 626 cases of appendiceal adenocarcinoma [20], which included 10 cases of pleura, 22 cases of lung, and 10 cases of both. Prognosis depended on histology. The authors concluded that lung metastasis from appendiceal adenocarcinoma may be higher than previously thought. It was not clear how many cases of PMP were included in the group of lung metastasis, but they may not be exceptional since appendiceal adenocarcinoma is frequently found to be ruptured at the time of laparotomy.

Pulmonary resection for pulmonary metastasis of colon cancer is recommended by NCCN guidelines [21], but few surgeons would consider surgical indication in cases with both pulmonary metastasis and peritoneal dissemination. PMP had been regarded as a noncurable disease for a long time, and repeated debulking surgeries were the choice of treatment [22]. Although CRS and HIPEC provided the possibility of cure as well as longer survival than conventional treatments, this aggressive locoregional cancer therapy is only performed at limited specialized centers and is still the subject of controversy at the majority of institutions. In addition, the rare incidence of PMP that was reported as one per million, the complexity of the procedures, and the high morbidity and mortality associated with the treatment are the main causes to prevent it from being accepted in general. The fact that there have been few reports of pulmonary metastasis from PMP may be related with the fact that CRS and HIPEC for curative intent were performed only at limited institutions and that, therefore, no interest was paid to pulmonary metastasis from PMP, which was noncurable by nature. The clinical implication of pulmonary metastasis from PMP would be more important if recognition that PMP can be cured by CRS and HIPEC become more popular in the future.

## 5. Conclusions

Extra-abdominal metastasis from PMP was exceptionally rare, but the lung was the most likely site in such a case. Clinical findings of PMP cases with pulmonary metastasis were similar to those without pulmonary metastasis. Resection of pulmonary lesions was indicated, and long survival may be expected when abdominal lesions were controlled by CRS and HIPEC. However, it should be cautiously noted that rapid progression after resection was seen in some cases, where biological features may be changed.

## Figures and Tables

**Table 1a:** (a)

Case	Reference	Sex	Age	Origin	Histology of origin	Abdominal surgery	Multiple/solitary	Laterality
1	Berge [4]	M	59	Appendix	Low grade	Palliative	Multiple	Bilateral
2	Chevillotte et al. [5]	M	45	Appendix	Low grade	Palliative	Multiple	Bilateral
3	Kreissig et al. [6]	F	39	Appendix	Low grade	Palliative	Multiple	Bilateral
4	Mortman et al. [7]	F	47	Appendix	Low grade	CRS + HIPEC^a^	Multiple	Right
5		M	48	Appendix	Low grade	CRS + HIPEC	Solitary	Left
6		M	41	Appendix	Low grade	CRS + HIPEC	Multiple	Right
7	Lee et al. [8]	M	60	Appendix	Low grade	CRS^b^	Multiple	Bilateral
8	Geisinger et al. [9]	M	61	Appendix	Low grade	CRS + HIPEC	Solitary	Right
9		F	45	Appendix	Low grade	CRS + HIPEC	Solitary	Right
10	Khan et al. [10]	M	65	Appendix	Low grade	Appendectomy + RT	Multiple	Bilateral
11	Present case (2012)	F	60	Appendix	Low grade	CRS + HIPEC	Solitary	Right

**Table 1b:** (b)

Case	Metachronous/ synchronous	Interval to pulmonary metastasis^c^	Pleural extension	Histology of lung	Pulmonary surgery
1	Metachronous	3 years	ND	Low grade	
2	Metachronous	7 years	(−)	Low grade	
3	Metachronous	5 years	ND	Low grade	
4	Metachronous	3 months	(−)	Low grade	Right lower lobectomy + LND
5	Metachronous	2 years	(−)	Low grade	Left lower lobectomy + LND
6	Metachronous	2 years	(−)	Low grade	Wedge resection
7	Metachronous	5 years	(+)	Low grade^d^	
8	Metachronous	ND	(−)	Low grade	Wedge resection
9	Metachronous	ND	(−)	Low grade	Wedge resection
10	Metachronous	7 years	(−)	Low grade	Right upper lobectomy, left upper lobectomy + wedge resection^e^
11	Metachronous	1 year	(+)	Low grade	Wedge resection

**Table 1c:** (c)

Case	Recurrence	Interval to recurrence	2nd surgery	Present status	Follow-up period^f^
1				DWD	
2				DWD	
3				ND	
4	(−)			NED	2 years
5	Abdomen	1 year	CRS + HIPEC	NED	3 years
6	(−)			NED	8 years
7				ND	
8	Lung	Shortly	No	ND	
9	(−)			NED	2 years
10	Lung	13 years	Wedge resection	NED	14 years
11	Lung + abdomen	2 months	No	DWD	1 year

CRS: cytoreductive surgery, HIPEC: hyperthermic intraperitoneal chemotherapy, RT: radiation therapy, ND: not described, DWD: died with disease, and NED: no evidence of disease. ^a^CRS + HIPEC were performed three times. ^b^The diaphragm was injured at CRS. ^c^Interval between the first abdominal presentation and the lung metastasis. ^d^Histology of the pleural lesion. ^e^Two-stage pulmonary resection was performed. ^f^Follow-up period from the pulmonary resection.
